# Long Island Veterinary Society

**Published:** 1890-05

**Authors:** D. S. Breslin

**Affiliations:** Secretary


### SOCIETY PROCEEDINGS.

*Long Island Veterinary Society.*—A regular meeting of the *Long Island Veterinary Society* was held on April 16th, 1890, at 74 Adams Street, the President, Dr. George H. Berns, in the chair. The following members were present: Drs. Berns, R. R. Bell, Bowers, Housman, Decker, Newman, Breslin, Atchison and Pendry. The minutes of the previous meeting were read and approved. The Board of Censors made no report. The Committee on Army Legislation, through its chairman, Dr. William H. Pendry, reported progress. Dr. Pendry also reported to the society that a bill was introduced and passed in the Assembly of the State of New York, granting an additional year to non-graduates to register in this State, and it is now awaiting the action of the Senate. He thought the society should take action upon the subject at once, and for that purpose he introduced the following resolution, which was adopted:

"That this society has learned with dismay that an act amending the act regulating the practice of veterinary medicine and surgery is now before the Legislature of this State, which amendment is for the purpose of granting a further extension of time in which non-graduates will be allowed to practice veterinary medicine, to the detriment of the profession who have qualified themselves in the interest of their profession and the public; and inasmuch as the present law was liberally framed for such non-graduates, and a period given in which they could register, and that time being twice extended by amendments to such act, this society protests against a further amendment for such purpose, as being unfair to the profession and calculated to work an injury to the public health, as by endless amendments to said act all inducements to qualify for the all-important work of the veterinary profession are removed."

The following committee of three was appointed by the chair on State legislation: Drs. William H. Pendry, R. R. Bell and George F. Bowers. This committee was appointed with full power, and they shall guard as much as possible the interests of the graduates and the profession in this State, and particularly oppose the extension of time as now proposed for non-graduates to register.

Dr. R. R. Bell read the following paper:

### EXAMINATION OF HORSES FOR SOUNDNESS.

I know of no branch of veterinary science in which there is more clashing of professional opinion, nor one upon which careful study and observation will yield more satisfactory results, than that very profitable department of our peculiar calling known as "Examination for Soundness." I can only give you a rough outline of the views of some of the many writers who have had occasion to discuss it, interspersed with a limited amount of original thought gained in the course of a short practice. I shall lead the discussion—for that is about all that is to be gained from the reading of this paper—and if by some remark I can excite some of the older heads to unbosom to us younger members some of the knowledge which long experience and acute perception have bestowed upon them, I will have accomplished all that I could have hoped for when I assumed the responsibility of undertaking so vast and so important a subject.

Each year I have become more and more convinced of the value of this branch of our profession to the community, and, judging by the increased demand for such services, I am led to believe that the public are imbued with the same estimate of it. Very few gentlemen in the large cities will now purchase a valuable animal upon their own judgment or upon the representations of the dealer. They want to know from a veterinary surgeon not only if a horse is sound, but they seek his opinion upon the conformation, disposition and general traits of the beast. I do not wish to be understood as saying that these latter points have anything in common with our duties; but I know that our clients invariably wish our judgment upon these matters. They lean upon us for guidance in their equine purchases, and often force us to an estimate of the horse's monetary value—a thing we should avoid wherever it is possible this side of actually offending our client. If the animal cannot pass a clean examination, they often make the issue definite by asking, in so many words: "Will he do my work?" They want to know if the horse is serviceably sound. I am sorry that this is so, for I am of those who do not believe that it is an easy matter to say of this horse, "He is sound;" of that horse, "He is unsound." This could be done if we had certain laws laid down for our guidance, certain things always constituting unsoundness, other defects to be classed simply as blemishes. We all know that a certain defect in one animal may render him unfit to perform work, while the same apparent trouble in another may be no worse than an eyesore. It requires a fine discretion, an acute judgment and long observation to do justice to the animal and to the buyer. Nothing makes more enemies for the faithful practitioner than examinations for soundness. A dissatisfied dealer can often prove to an intending purchaser that his veterinarian's opinion is valueless by procuring an opinion from an equally reliable source, having a contrary verdict upon the animal's bodily condition, and each certificate may be honestly given based upon conscientious belief. This comes from the fact that different men view the animal from different standpoints, and estimate differently upon certain conformations and conditions. A writer in the March number of the *Veterinary Journal* exemplifies this assertion by pointing out that a man who has more particularly devoted himself to the study of physiology would be more inclined to first familiarize himself with the state of the animal's health; he would inquire into the condition of the circulatory apparatus, by noting its effects upon the visible mucous membranes, observing the character and speed of its movements as denoted by the pulse; he will auscultate the respiratory murmur, and note any physiological or structural abnormalities there to be met. He will look into the functional activity of the principal glands of the system; carefully take note of the character of the intestinal secretory glands, by watching the condition of the fæces; of the sudoriferous and sebaceous glands, by observing the effects of exercise for the former, and examining the condition of the coat for the latter. He obtains an idea of the quantity and quality of the renal excretion to speak for the condition of the kidneys. He will not omit to make a thorough examination of the organs of special sense, not only viewing the eye from every angle, but will bring his ophthalmoscope into requisition to determine if there is complete transparency of the crystalline lens, if the vitreous humor is devoid of foreign floating bodies, if the retina is luminous and normal, and if there is turgescence of the choroidal vessels. But, on the other hand, we find a man, equally as conscientious, though more practical, who deems these details, if not absolutely non-essential, of very minor importance. He is spending his time in a much more practical way. He moves about the animal with a studied grace, and touches with a systematic and theatrical show and grave composure, his educated hand making a few quick strokes of the neck, down the front legs, over the fetlocks; slipping back to the rear, he dexterously manipulates the hips and the hocks. He brings down the admiration of his audience, of the usual stable contingent, by a single glance at the incisor teeth, a fillip of the fingers in front of the eye, to test the visionary powers of the wondering beast, and, with a dig in the side, he is prepared to pronounce the animal not a "roarer." With a trot up and down the street, he is then ready to give his opinion to the owner as to the soundness of the animal. So these two men may observe the same defect, but it will have a different significance to each in many cases.

Again, personal experience with certain structural affections will influence an individual estimate of their significance. Those who have never met a case of side bone producing lameness would be disposed to undervalue its importance, while a practitioner observing hygroma of the hock only as a blemish would not feel justified in sending its possessor back to his master; or he who has only found splints cool and located on the rudimentary metacarpal bone, would hesitate in dealing harshly with one situated a little anteriorly. But, conversely, an examiner who is fresh from a poor result in treating such an affection as any of the foregoing would pronounce severe judgment on such abnormalities. So there are very many reasons why two practitioners examining the same animal may differ in their estimate of the horse's worth.

We will not speak to-night of that veterinarian who becomes so expert with his "practiced eye" and "educated touch" that a careful examination is not necessary for a thorough knowledge of the horse's condition. The time has passed when a hurried glance over the stall door will reveal to the intuitive mind of the "born veterinarian" a perfect insight into the anatomical structure and physiological functions of the salable soliped. We have no patience with a man who can see in his "mind's eye" this same horse ten years hence performing his work to the perfect satisfaction of his trusting client.

But, to the careful, painstaking, intelligent veterinary surgeon, who goes about his task in a systematic, professional manner, allowing no point to escape him, testing every function, and using every precaution to avoid overlooking any defect, carefully weighing everything which has a bearing upon a correct and conscientious opinion upon the condition of the animal he is called upon to examine, we desire to address ourselves, and try to assist him by an interchange of views here in this society's hall. Our time is too limited to discuss the many definitions of "soundness" which are as various as the writers upon the subject are numerous. I am not willing to deny that the definition given us many years ago by Mr. Percival is not as good as any that have preceded or succeeded his contribution to veterinary jurisprudence—so far as it goes. He says: "Any horse which is lame, or has that about him which is likely to render him lame, is unsound." But surely, a horse may be absolutely unsound, and yet free from lameness. I should not employ the second time a doctor who allowed me to purchase an animal affected with pulmonary emphysema, simply because he was not lame. Professor Liautard makes a decided improvement upon this qualifying definition when he says: "An animal to be sound must be as near perfection as possible; must be free from diseases likely to render him useless. We may find remains of disease, and yet he may be perfect enough to be a useful animal." I do not like that portion of this definition which says "likely to render him useless." He need not be useless to be unsound. He may be able to be useful, and yet his usefulness may be of an unsatisfactory character; and if we find "remains of disease and yet a useful animal," he is not necessarily a sound one. Possibly we may be able to arrive at a definition later on which will be the legal standard the world over. To examine an animal thoroughly we should see him at rest and in action; we should see him hot and see him cold When at rest in the stall, we are permitted to watch how he deports himself, what he does and how he does it; and especially should we note his first movements when being backed out into the gangway, for I need not tell you the significance of the symptoms which may here be displayed. Examine him in the full light. Place him squarely on his legs, devoid of every vestment save the halter. Look at him from every side, carrying in your mind whatever irregularities you may detect. From this position there are many points which you will be enabled to note. You will measure with your eye how he stands upon his legs, their contour, and the conformation of important joints and sections; in a minute you have seen if there exists a curb, if he knuckles at the fetlocks, if he "goes over" at the knees, and many other points of value to be remembered when you begin to look into the details. The more experience one has the more he becomes impressed with the value of a system in examinations. Begin at one point and dissect the live body with care and with system, using all of your special senses Starting at the labial muscles, examine everything of importance about the head, mouth, age, pulse, intermaxillary space, eyes, ears, parotid gland and temporal fossa. Passing down the neck, mark its junction with the thorax, the withers, the shoulders, the anterior extremities, the vertebral column, the chest, trunk; then do for the hind legs what you have done for the front ones, the hips, the stifle, the hock, and so on to the foot. Examine the inguinal region, feeling the condition of the scrotum and terminating with the tail and anus.

Your animal may have stood this examination with credit, and now you order him to be taken into the street. Though he may have shown no unsoundness at rest, he may develop it when thrown into action. He should be led by the halter, with possibly a foot of liberty, and handled by a hostler who knows his business. He should be trotted toward you, away from you and in front of you, and displaying a regularity of action which only comes from the perfect working of every joint. If the defect be not in his gait, it may exist in his respiratory apparatus; or there may be abnormalities in his circulatory system. And we should not consider any examination where palpable unsoundness has not been found complete until the horse has been cooled off, after having been well heated up, and again submitted to an examination in action. Many imperfections are brought out thus which otherwise would escape our observation.

Now what will we look for to constitute unsoundness? As stated, we will begin at the head, and look for malformations or diseases, employing all our special senses to help us. You are standing directly in front of the animal and you will note his age, which will display the natural or the artificial incisors; if he is a crib-biter, necrosis of the inferior maxilla at the interdental space or caries of the teeth. Peering into the nostrils, we may find the pathological lesions of acute or chronic inflammation of the Schneiderian membrane, suppuration of the maxillary sinuses, necrosed bones, the ulcerations of glanders, or the petechiæ of purpura hemorrhagica. Dropping the hand into the intermaxillary space, we feel its bony walls for evidences of osteo-porosis or other diseases or abnormalities; and running along the bottom of the hyoid space our hand may come in contact with the hot and painful fluctuating abscess of rhino-adenitis, or the indurated, nodulated adherent swelling of glanders. While stopping a moment here you have an opportunity of getting the movement of the pulse as the glosso-facial artery turns around the rami of the inferior maxilla. Standing there we will examine very carefully each eye, comparing one with the other, and if our ocular inspection be not absolutely satisfactory the ophthalmoscope will be indispensable, especially so if there is suspicion of periodic ophthalmia, noting the changes in the lens, the vitreous humor and the circulation.

Mounting to the occiput, one finger will slip into the ear for foreign bodies or neoplasms, and pass back over the poll for fistula or the cicatrix of a past one.

The hand then seizes the larynx in search of anatomical imperfections, and by a gentle squeeze we test its walls for ossification of the cartilages; and at the same time will be rewarded by an involuntary cough, which may reveal much that will assist us in determining the physiology of the lungs and throat. A look at the parotid region may reveal marks of setons, cicatrizations of evacuated abscesses or traces of blisters, which would give you the history of a past laryngitis. Pressure of the finger in the jugular groove will dam the blood in a normal vein, and if the dilatation be bassilated in character we would suspect that our animal had been the subject of repeated bleeding, which may have been necessitated by such an unwonted affection as head staggers; or if dilatation does not take place, our vein may have become obliterated from previous phlebitis. It may be that the trachea is irregular or angular, which might have been produced by fracture of a ring, or from the operation of tracheotomy, which we know is often followed by the complication of roaring. It would not be out of place here to feed the animal just enough to determine the absence of a jabot, and to see if mastication and deglutition are performed in a natural manner.

The hand and eye passing carefully over the withers would convince us of the absence of fistula, cysts, abscess, or deceased bone in this region, and keeping on down the vertebral column, and pressing with the fingers, on arriving at the lumbar region we would expect to see our animal yield gently, as do all horses who are enjoying immunity from renal affections, or whose spinal bones have not become ossified. A gross inspection of the sides of the chest may show marks of blisters, the rhythm of respiration, or the double movement of heaves. The ear placed against the thoracic walls will give the music of the vesicular murmur, as the air cells fill in the whole extent of the surface of the lungs.

At the umbilicus we may find marks of hernia, and at the inguinal opening we look for the complications of castration, or, if he be a stallion, our mind would be at rest if we made a rectal exploration and found the internal openings of the canal alike on each side, thus doing away with the possibility of his being subject to intermittent hernia.

By scrutinizing the penis or vulva, the tail and the anus, we are then prepared to go into a detailed examination of the extremities.

As a general rule, any lame animal is unsound; and, as a rule, we will not discuss whether the lameness is acute or chronic. If he is lame at the time we examine him, he is unsound. There are many pathological lesions and structural changes found upon the legs of horses which will not interfere with their action, and it is just here that judgment, reason and experience will stand us in well. It would be unjust to condemn horses for defects which we know will not interfere with their usefulness or their perfect performance of the work exacted of them.

First, we will examine the forelegs, and in doing so we will not be hasty. We will observe his general mode of standing, whether his legs are on a plumb-line, in advance of it, or behind it; if too wide apart, or if the elbows are abducted. At the scapulo-humeral articulation we would look for traces of setons or blisters, giving us the history of a past lameness; and while examining here, the hand drawn over the spinatus fossa and shoulder joint, would discover small tumors from collar pressure. Possibly we have an atrophy of the muscles of this section or malformations in the scapular region. The arm will very seldom be found the seat of the disease, but at the point of the elbow we find cysts or neoplasms, which are unsightly if not unsoundnesses.

Arriving now at the forearm and knee-joint, we scrutinize more closely, to reinforce the opinion formed while viewing him from the side, and are careful to note if there is a tendency to weak knees, if not absolutely "sprung;" and while manipulating this region we may find on the inside of the carpal joint an osseous or fibrous thickening, reminding us that at some time the animal has inflicted speedy cuts or blows, and storing this point away, we are careful to note his manner of travelling, to see if he has overcome that interference. Around the front of the knee the hand may feel a cicatrix, from broken knee, telling us how, at some time, he had fallen upon them, but not necessarily denoting that he is a stumbler. We will find here, too, enlarged bursæ, constituting a carpal thoroughpin. We now have descended to the digital region, and along the metacarpals we find splints, which are unsoundnesses in some cases, calling into functional activity the examiner's judgment and knowledge. At the back of this region we find the important tendons which bear the whole weight of the anterior two-thirds of the trunk at certain times, and are very liable to disease, and should we discover sprains of the tendons, ligaments or sheaths, or indurations from previous inflammations, we know that sooner or later they will give away, and the animal will not conform to our definition of soundness.

At the fetlock we will find dilatations of tendinous and articular bursæ. The region below the fetlock is fraught with many defects, and requires the closest scrutiny and judgment, for here we find that most serious lesion, ringbone, the simple presence of which requires an unqualified condemnation. Here, too, we will find on the inside marks of old or recent interfering, while just above the fetlock, on both the inside and outside, may be found the small cicatrices of neurotomy, or possibly the low operation may have placed the scars below the fetlock. Careful examination of the front foot is one of the most essential points in the examination, and it is best tested by the hoof-searching forceps for bruises or corns or weak soles, while with the hand we will examine for side-bones, and, having the foot raised from the ground, we note if the plantar surface is flat or pumaced, if the heels are contracted, if toe or quarter cracks be present, and our examination is not complete if we have not looked for thrush or canker. All of these diseases or conditions are likely to produce lameness, and our judgment must be formed according to our knowledge of their condition when making the examination.

Passing now to the rear in our detailed examination, we glance at the general position of the animal—from the hip to the thigh on each side, noting variations in the size of the two members, the shape of the hocks and the obliquity of the fetlocks. Standing behind the animal, with one hand on each hip, looking first at the external angle of the ilium on one side, then the other, noting if they are on a level with each other and at equal distances from the sacrum. Here, too, we may find atrophy of the gluteal muscles of one side—not an unsoundness of itself, but possibly connected with former lameness; at the coxo-femoral joint evidences of counter-irritation having been applied to overcome positive or fancied diseases of this region. Passing the hand down the femur, we reach the stifle, and, failing to find anything abnormal, we proceed to the hock. We will remember, just here, that a healthy hock consists of skin, bone and a few tendons; anything superfluous is abnormal. At the internal and anterior aspect of the region we may discover a dilatation of the synovial membrane of the tibio-tarsal articulation, constituting the ordinary blood or bog spavin, or the lesion may be of the tendinous sack of the tendo-Achilles, forming a thoroughpin. At the posterior surface we will look for exostoses and curbs. There is as little unanimity of opinion among veterinarians upon the soundness of horses possessing these defects as there exists among astronomers as to the distance of the furthest fixed star. It is, of course, unnecessary for me to speak of exostoses on the internal aspect of the hock joint, as I think the profession is a unit in condemning every animal possessing such an addition to his anatomy. Personally, I not only reject an animal when they are present, but will not tolerate the suspicion of such a condition. There is another thing to be carefully watched for, and that is the presence of stringhalt. Whatever the pathological lesion may be, it is an unsoundness, and it is sometimes so slight in its incipiency that we may overlook it. Therefore the first movements of the animal in backing out of the stall are of special importance to us, and a quick turn of the animal may produce the symptom where it is latent in the forward movements.

If chronic scratches of the front of the hock is not an unsoundness, it is a great nuisance, and it should be condemned, along with chronic greasy heels. Below the hock we have about the same conditions as met with in the anterior extremity, only that interfering is better characterized. Puffy swellings may exist at the fetlocks, and the foot should be examined, especially to note if shod for interfering or over-reaching.

For this hurried and imperfect paper I must make same apology, but I am so sure that I have said a few things which are not in accord with the views of many present, that I feel you will be amply repaid by the expostulations and corrections of outspeaking minds.

The reading of the paper was followed by quite a discussion, after which a vote of thanks was unanimously tendered to the essayist. It was moved by Dr. Bell, and seconded by Dr. Bowers, that the secretary have two type-written copies of the proceedings of the society made, and forward one each to the *American Veterinary Review* and JOURNAL OF COMPARATIVE MEDICINE AND VETERINARY ARCHIVES. Carried.

Moved by Dr. Bowers, and seconded by Dr. Newman, that the Secretary order fifty reprints of the proceedings of the society from the editor of the *American Veterinary Review.* Carried.

The Secretary reported that he had communicated with Dr. R. A. McLean, as directed, in regard to the return of Dr. R. R. Bell's paper on "Azoturia," and had failed to receive a reply from the gentleman up to the present time.

The chair appointed as essayist for the May meeting Dr. Samuel Atchison.

The meeting then adjourned.

D. S. BRESLIN,
*Secretary.*

